# Detection of Postictal Generalized Electroencephalogram Suppression: Random Forest Approach

**DOI:** 10.2196/17061

**Published:** 2020-02-14

**Authors:** Xiaojin Li, Shiqiang Tao, Shirin Jamal-Omidi, Yan Huang, Samden D Lhatoo, Guo-Qiang Zhang, Licong Cui

**Affiliations:** 1 Department of Neurology University of Texas Health Science Center Houston, TX United States; 2 Department of Computer Science University of Kentucky Lexington, KY United States; 3 School of Biomedical Informatics University of Texas Health Science Center Houston, TX United States

**Keywords:** epilepsy, generalized tonic-clonic seizure, postictal generalized EEG suppression, EEG, random forest

## Abstract

**Background:**

Sudden unexpected death in epilepsy (SUDEP) is second only to stroke in neurological events resulting in years of potential life lost. Postictal generalized electroencephalogram (EEG) suppression (PGES) is a period of suppressed brain activity often occurring after generalized tonic-clonic seizure, a most significant risk factor for SUDEP. Therefore, PGES has been considered as a potential biomarker for SUDEP risk. Automatic PGES detection tools can address the limitations of labor-intensive, and sometimes inconsistent, visual analysis. A successful approach to automatic PGES detection must overcome computational challenges involved in the detection of subtle amplitude changes in EEG recordings, which may contain physiological and acquisition artifacts.

**Objective:**

This study aimed to present a random forest approach for automatic PGES detection using multichannel human EEG recordings acquired in epilepsy monitoring units.

**Methods:**

We used a combination of temporal, frequency, wavelet, and interchannel correlation features derived from EEG signals to train a random forest classifier. We also constructed and applied confidence-based correction rules based on PGES state changes. Motivated by practical utility, we introduced a new, time distance–based evaluation method for assessing the performance of PGES detection algorithms.

**Results:**

The time distance–based evaluation showed that our approach achieved a 5-second tolerance-based positive prediction rate of 0.95 for artifact-free signals. For signals with different artifact levels, our prediction rates varied from 0.68 to 0.81.

**Conclusions:**

We introduced a feature-based, random forest approach for automatic PGES detection using multichannel EEG recordings. Our approach achieved increasingly better time distance–based performance with reduced signal artifact levels. Further study is needed for PGES detection algorithms to perform well irrespective of the levels of signal artifacts.

## Introduction

### Background

Epilepsy is one of the most common neurological disorders, and it affects an estimated 65 million people worldwide [1]. An epileptic seizure (hereafter referred to as seizure) is a brief episode, usually with signs or symptoms because of transient, undesired, excessive, and synchronous electrical discharge, involving large numbers of neurons in the brain [2]. When seizure occurs, altered movement, expression, and levels of consciousness are often observed in the affected person. Seizure may produce temporary confusion, uncontrollable jerking movements of the arms and legs, inability to speak, or loss of consciousness or awareness [3].

In a worst-case scenario, frequent seizures may predispose a person to sudden unexpected death in epilepsy (SUDEP) [4]. Among neurological events and conditions, SUDEP is second only to stroke in years of potential life lost, highlighting the importance and significance of this condition for public health [5]. SUDEP is a catastrophic and fatal complication of epilepsy. The definition of SUDEP is “sudden, unexpected, witnessed or unwitnessed, non-traumatic and non-drowning death, occurring in benign circumstances, in an individual with epilepsy, with or without evidence for a seizure and excluding documented status epilepticus, in which postmortem examination does not reveal a cause of death” [6], that is, no other cause of death can be found [7]. However, the mechanisms underlying SUDEP are not completely understood.

Electrophysiological signals such as electroencephalogram (EEG), electrocardiogram, and electromyography, collected together in the epilepsy monitoring unit (EMU), are traditionally used for understanding epileptic seizures [8]. Noninvasive scalp EEG and invasive intracranial EEG are the most commonly used methods for locating seizures and monitoring the interphase activity between seizures [9]. Invasive intracranial EEG is one of the techniques used in localizing the seizure onset zone in preparation for surgery [8]. EEG is a key source of information for the diagnosis of epilepsy, including whether epilepsy is focal or generalized, idiopathic or symptomatic, or part of a specific epilepsy syndrome [10]. Therefore, EEG has also been widely used to identify biomarkers that can help prevent the development of epilepsy, identify focal brain regions that produce epilepsy, and ultimately cure epilepsy through surgical means [11].

Postictal generalized EEG suppression (PGES) is a potential EEG biomarker of SUDEP risk [12-14]. PGES is a period of brain inactivity after seizure. It most often occurs after generalized tonic-clonic seizures (GTCS), particularly in those arising from sleep, and is related to the symmetric tonic phase, postictal immobility, lack of early oxygen administration, duration of oxygen desaturation, and lower peripheral capillary oxygen saturation nadir values [15-17]. GTCS are the most significant risk factor for SUDEP [13]. PGES is defined as a diffused EEG background attenuation (<10 μV) in the postictal period [18]. Prolonged PGES (>50 seconds) has been reported in refractory epilepsy patients who are at risk of SUDEP [14]. For every prolonged second in the duration of PGES, the odds of SUDEP is increased by a factor of 1.7% (*P*<.005) [14].

**Figure 1:**
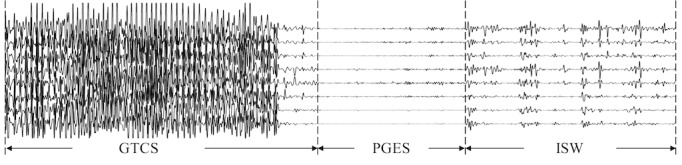
An example of postictal generalized electroencephalogram suppression and intermittent slow wave activity signals after generalized tonic-clonic seizures. GTCS: generalized tonic-clonic seizures; ISW: intermittent slow wave; PGES: postictal generalized electroencephalogram suppression.

**Figure 2:**
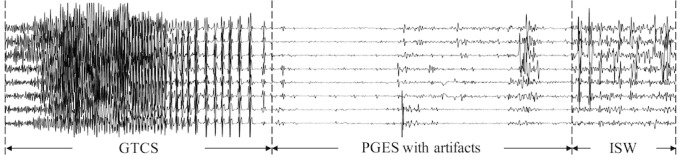
An example of postictal generalized electroencephalogram suppression and intermittent slow wave activity signals (with artifacts) after generalized tonic-clonic seizures. GTCS: generalized tonic-clonic seizures; ISW: intermittent slow wave; PGES: postictal generalized electroencephalogram suppression.

Clinically, the determination of the duration of PGES is manually performed by human experts through visual inspection of EEG signals. According to definition, the identification of PGES appears to be straightforward by identifying a period of low-amplitude EEG signals after the seizure, as shown in Figure 1. However, real-world data recorded in EMUs may contain high-amplitude signals caused by physiological artifacts (eg, breathing, muscle, and movement artifacts), as shown in Figure 2. Therefore, clinical experts usually leverage additional video recordings along with signals to identify high-amplitude artifacts that are not real EEG activities [19]. Automated PGES detection tools are highly desirable to assist clinical personnel in reviewing and annotating PGES in EEG recordings. Automated techniques have been extensively studied for epilepsy-related EEG signal analysis [20], including a random forest classifier with empirical wavelet transform for seizure identification [21], a data-driven approach for classifying seizure and nonseizure EEG signals using the multivariate empirical mode decomposition algorithm [22], a whole-brain seizure detection approach using the K-nearest neighbors classifier [23], and extreme epileptic events detection and prediction using neural networks with time-frequency features [24,25]. However, there has been only one study [19] using logistic regression to perform automated PGES detection based on frequency-domain features of EEG signals. The following challenges remain in developing a fully automatic PGES detection tool:

- The presence of artifacts remains the main challenge that makes PGES detection more complex than applying a fixed amplitude threshold.
- There is no sensitive and standardized criterion dedicated to measuring and evaluating the performance of PGES detection algorithms.

In this paper, we introduce a random forest–based classifier for PGES detection by leveraging a variety of EEG signal features such as time-domain features, frequency-domain features, wavelet-based features, and interchannel correlations. We incorporate confidence-based correction rules to remove suspicious sudden changes of EEG activities. This study focused on identifying the first slow wave brain activity, that is, the onset of the first intermittent slow wave (ISW) activity (see Figure 1 and Figure 2), which indicates that the brain activity will gradually recover [12,14]. Therefore, the output of our PGES detection method for each signal recording is the onset of the first ISW. Accordingly, traditional segment-based performance evaluation methods are not well suited for PGES detection. Instead, we introduced a new, recording-by-recoding evaluation method dedicated to PGES detection with direct practical relevance.

### The Center for Sudden Unexpected Death in Epilepsy Research

The Center for SUDEP Research (CSR) is a National Institute of Neurological Disorders and Stroke–funded Center Without Walls initiative for collaborative research on epilepsy. It comprises researchers from 14 institutions across the United States and Europe, bringing together extensive and diverse expertise to understand SUDEP [4,26]. The goal of CSR is to better understand cortical, subcortical, and brainstem mechanisms responsible for SUDEP and to use a data-driven, systems biology approach to elucidate the role of cortical influences in SUDEP. To advance SUDEP research, CSR created an infrastructure to fully, effectively, and efficiently utilize a range of prospectively collected data from different domains, including clinical, electrophysiological, biochemical, genetic, and neuropathological fields. CSR provides a comprehensive, curated repository of prospectively collected multimodal data, including electrophysiological signals in European data format. These data are linked to risk factor and outcomes data of over 2500 epilepsy patients (a broad spectrum of ages as well as social, racial, and ethnic groups) with thousands of 24-hour recordings.

### Feature Extraction From Electroencephalogram Signals

For EEG signal feature extraction, the following 4 categories of features are considered in this work: (1) time-domain features, (2) frequency-domain features, (3) wavelet-based features, and (4) interchannel correlations.

1. Time-domain features: Time-domain features include statistical measures and Hjorth parameters. Statistical measures include nth percentile of the signal, average, range, standard deviation, skewness, and kurtosis [27]. Here, the mean measures the central tendency, skewness measures the asymmetry, and kurtosis measures the tailedness of a probability distribution. Hjorth parameters are commonly used for feature extraction to perform EEG signal analysis [28], including mobility and complexity [29-31]. The mobility represents the mean frequency or the proportion of the standard deviation of the power spectrum. The complexity indicates the signal’s similarity to a pure sine wave [31].
2. Frequency-domain features: An EEG wave (captured by an electrode) comprises many other waves with different amplitudes and frequencies. Therefore, an EEG signal has different bands, defined by the frequency of the waves, such as slow oscillations (0.5 Hz-1 Hz), delta bands (1 Hz-4 Hz), theta bands (4 Hz-8 Hz), alpha bands (8 Hz-12 Hz), beta bands (14 Hz-30 Hz), and gamma bands (30 Hz-80 Hz). The spectral power in a specific frequency band, for instance, the 0.5 Hz to 1 Hz band, can be regarded as a feature.
3. Wavelet-based features: Wavelets are a relatively recent approach for signal processing, and the main advantage is that wavelets allow multiresolution analysis in time and frequency simultaneously [32,33].
4. Interchannel correlations: Many studies have attempted to find movement-related information of connectivity between different brain regions [34-38]. Correlation analysis represents the degree of relatedness and synchrony between 2 time series. It indicates similar information as a cross-coherence analysis of different EEG channels [39].

### Random Forest

Random forest is an ensemble learning method used for classification and regression problems. This method has been used for automated sleep stage classification based on EEG signals [40,41]. Random forest involves a group of decision trees during training and outputs the mode of the classes predicted by individual trees. The overall output is determined by applying the input to each tree and choosing the class that gets the most weighted vote. The weight of each tree is adjusted using misclassification and out-of-bag measures.

As it is different from other traditional classifiers (eg, K-nearest neighbor, support vector machine, and artificial neural network), we select random forest as the approach for our study because of the following advantages: (1) adaptable, as it estimates the importance of variables and provides a way for tuning with additional training data by assigning different weights for each decision tree; (2) scalable, as it can handle thousands of input variables and work efficiently on large datasets; and (3) robust, as it can balance errors in datasets with unbalanced class population [41].

## Methods

### Overview

The dataset used for this study comprises 116 EEG signal recordings from 84 patients with GTCS in the CSR data repository, with PGES annotated by domain experts. We extracted the 5-min postictal EEG signals for PGES detection. A total of 8 EEG channels are utilized: Fp1-F7, F7-T7, T7-P7, Fp2-F8, F8-T8, T8-P8, Fz-Cz, and Cz-Pz.

**Figure 3:**
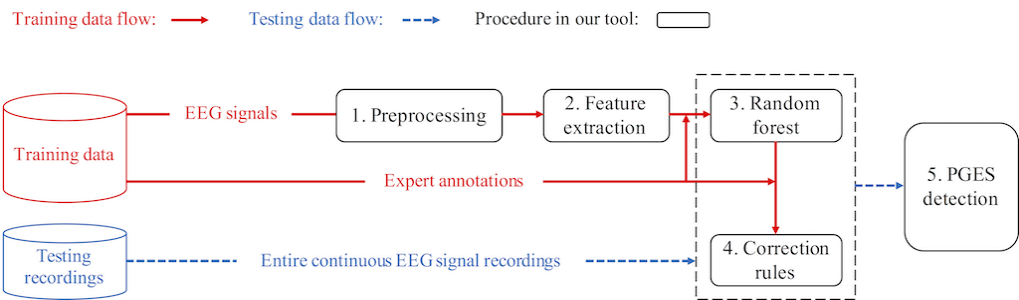
The overall workflow of our automated postictal generalized electroencephalogram suppression detection approach. EEG: electroencephalogram; PGES: postictal generalized electroencephalogram suppression.

The overall workflow of our PGES detection method is shown in Figure 3. The process started with the preprocessing of the EEG signals (step 1), followed by feature extraction (step 2). Then a random forest classifier was trained and tested based on the extracted features (step 3). We applied correction rules, which are constructed based on the continuity of brain activities, to the prediction of the random forest (step 4) and provided the final detected label for each signal segment (step 5).

### Preprocessing

Each postictal EEG signal record is split into signal segments with a length of 1 second (ie, 1-second epoch) from the beginning to the end without overlapping. The common electrophysiological artifacts present in the EEG signal recordings include muscle artifacts, breathing, and body and bed movements [42]. The main frequency of ISW is less than 5 Hz. To minimize the presence of residual artifacts, the signals are filtered with a band-pass filter with cutoff frequencies at 0.5 Hz and 5 Hz.

### Feature Extraction

**Figure 4:**
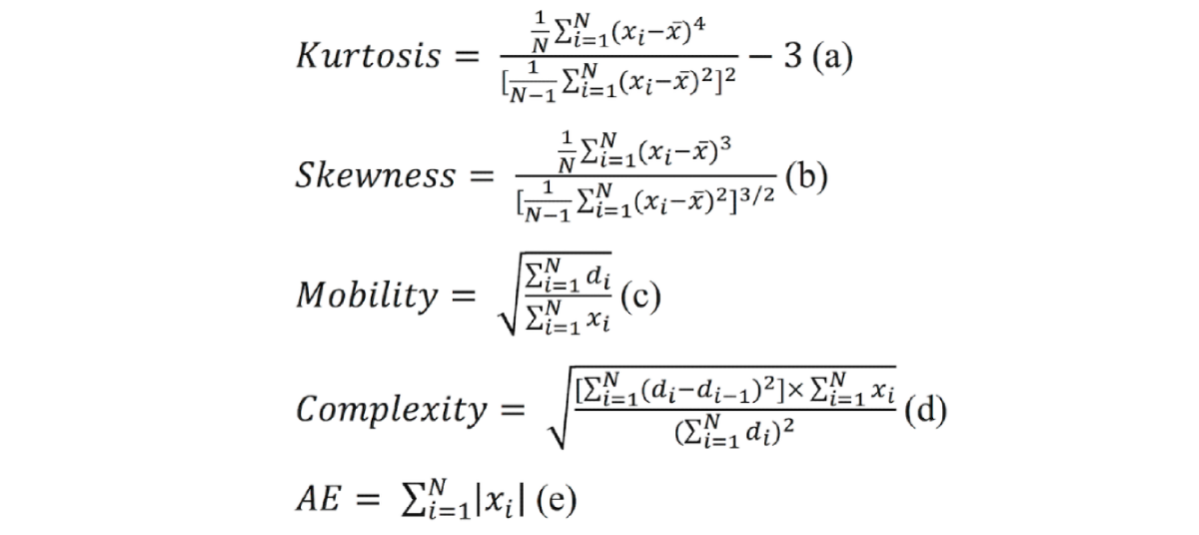
The definitions of the time-domain properties. AE: amplitude energy.

For each signal segment of the 8 EEG channels, we extracted 76 features including time-domain features, frequency-domain features, and wavelet-based features as follows:

- The following 16 time-domain features were extracted: (1) 11 statistic features, including mean, median, maximum, minimum, range, standard deviation, *n*th percentile of the signal (*n*=5, 25, 75, and 95), and the root mean square, and (2) 5 time-domain properties of kurtosis, skewness, mobility, complexity, and amplitude energy (AE) of the signal. The time-domain properties for a time series *X={x_1_, x_2_, ..., x_n_}* are defined in Figure 4 [27-30], where *N* is the number of data points, 
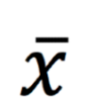
 is the mean of *X*, and *d_i_=x_i_−x_i_−_1_ and i=1, ..., n*.
- A total of 4 frequency-domain features were extracted. As the PGES and ISW are typically low-frequency brain activities (0.5 Hz-5 Hz), we extracted the spectral power of 4 low-frequency bands consisting of 0.5 Hz to 1 Hz, 1 Hz to 2 Hz, 2 Hz to 4 Hz, and 4 Hz to 5 Hz.
- A total of 56 wavelet-based features were extracted. EEG signals were subjected to three-level decomposition using the Daubechies 4 wavelet. From the decomposition process, a total of 4 coefficient sets were generated, and we calculated 14 measurements (except range and AE) used in time-domain features for each coefficient set as wavelet-based features [32,33].

**Figure 5:**
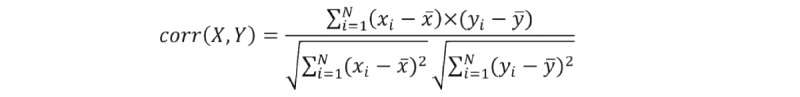
The definitions of the linear correlation coefficient between two time series (X and Y).

To capture cross-coherence of EEG channels, we further investigated the interchannel correlations using the linear correlation coefficient between selected channels. The correlation coefficient for 2 time series, *X={x_1_, x_2_, ..., x_n_}* and *Y={y_1_, y_2_, ..., y_n_}* is defined in Figure 5 [39], where *N* is the number of data points and 
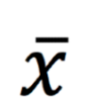
 is the mean. For each signal segment, we calculated 4 interchannel correlations: *corr(Fp1−F7, Fp2−F8)*, *corr(Fp2−F8, Fz−Cz)*, *corr(Fp1−F7, Fz−Cz)*, and *corr(Fz−Cz, Cz−Pz)*.

### Random Forest Classifier

There are 5 steps to build the random forest classifier with the bootstrap aggregating (bagging) technique [43], which is an ensemble method to reduce the variance without increasing the bias for decision tree algorithms. Given a training set *X={x_1_, x_2_ ,..., x_n_}* and *Y={y_1_, y_2_, ..., y_n_}*, the 5 steps are as follows:

1. Generate a subtraining set *S={X_s_, Y_s_}* by selecting a random number of observations and features from the whole input training dataset.
2. Build and train each decision tree *T_i_* (eg, regression tree, random tree, and C4.5) with the generated subtraining set *S*. In the construction of each decision tree, nodes and leaves are built by selecting a random number of features. This process will minimize the correlation among the features and decrease the sensitivity to noise [40].
3. Estimate out-of-bag errors. In the training process of each tree, about two-thirds of *S* are used for tree construction, and the remaining one-third is used to test the classification performance of the tree. Therefore, it gets an unbiased estimate of the test set error internally in a random forest, and there is no need to use further cross-validation [43].
4. Repeat the above steps (step 1, step 2, and step 3) *N* times to build *N* decision trees *T={T_1_, T_2_, ..., T_N_}*.
5. Compute the classifier output. After training, the output *y'* for an unknown sample *x'* can be made by averaging the output from all the individual decision trees on *x'*:

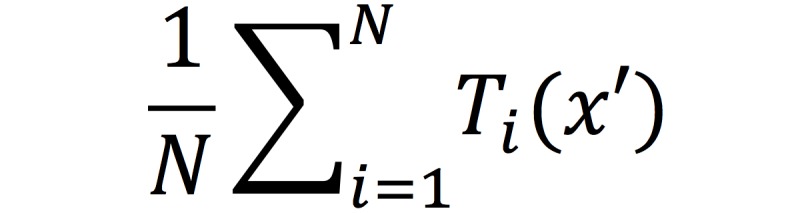

This classifier can obtain strongly correlated trees by training all trees with the same training set, and bagging is a way to decorrelate the trees. The prediction results of a single decision tree may be highly sensitive to noises in its particular training set, especially with overfitting. However, in a random forest, the average of all trees is less sensitive to noises as the trees are more decorrelated. In this work, we used a random forest with *N=1000* trees.

#### Correction Rules for Continuous Detection

According to the knowledge of the domain experts, longtime EEG suppression does not often occur after the first ISW happens in practical scenarios. This indicates that sudden changes of PGES/ISW states are unlikely to happen. For example, for a sequence of predicted labels with 10 consecutive 1-second segments (PGES, PGES, PGES, ISW, PGES, PGES, PGES, PGES, PGES, and PGES), the sudden changes from PGES to ISW (from the third segment to the fourth segment) and from ISW back to PGES (from the fourth segment to the fifth segment) are unlikely, that is, the predicted label for the fourth segment is most likely a misclassification and should be corrected and replaced with PGES. Therefore, we considered the temporal contextual information of segments to perform correction.

We constructed confidence-based correction rules based on the probability output of the random forest classifier. For each segment *Seg_i_*, we built a confidence index, *conf(Seg_i_)* based on the average probability from the current segment to the next *M* segments; the definition is as follows:

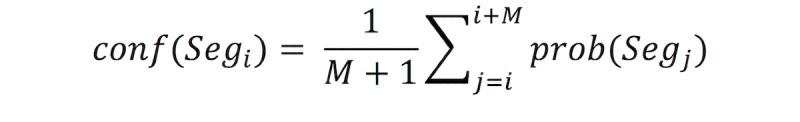



where *prob(Seg_j_)* is the random forest probability output of the segment *Seg_j_*.

In this work, we chose *M=5*. On the basis of the probability and confidence index of each segment, we applied the following 3 rules to correct suspicious sudden changes of PGES/ISW states in the detected label sequence:

1. If *prob(Seg_i_)* had a high value but the probabilities of *Seg_i_’s* surrounding segments (eg, *prob(Seg_i-1_)* and *prob(Seg_i+1_)* had low values, then we corrected the detected label of *Segi* as PGES.
2. If *conf(Seg_i_)* had a high value but the confidence indexes of *Seg_i_’s* surrounding segments (eg, *conf(Seg_i-1_)* and *conf(Seg_i+1_)*) had low values, then we corrected the detected label of *Seg_i_* as PGES.
3. If *prob(Seg_i_)* had a high value but *conf(Seg_i_)* had a low value, then we corrected the detected label of *Seg_i_* as PGES.

#### Performance Evaluation

**Figure 6:**
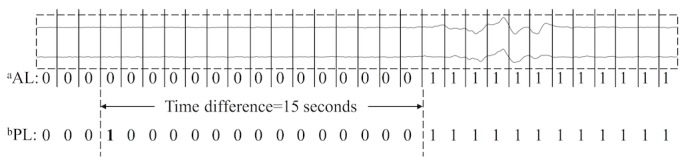
An example of automatic postictal generalized electroencephalogram suppression detection evaluation. Annotated labels are the expert-annotated labels, and predicted labels are generated using the automatic detection algorithm. ^a^AL: annotated label; ^b^PL: predicted label.

For traditional EEG signal classification tasks such as sleep stage classification [40,44,45], the performance evaluations are segment-based (ie, the predictions for each segment determine the performance metrics such as accuracy, precision, and recall). However, for the PGES detection setting, the prediction result of the onset of the first ISW in a given signal recording (ie, recording-based) is more important as it indicates the end of PGES. In other words, the prediction results of segments after the first ISW become less important for the evaluation. Figure 6 shows an example of the signal that is split into 30 segments of 1-second each, and each segment is annotated with a label *0* for PGES or *1* for ISW (see the annotated labels). The predicted labels are generated by the automatic detection method; the predicted label highlighted in bold means that the label is wrongly predicted, whereas other labels are correctly predicted. In this example, only one label is wrongly predicted, indicating that the PGES detection method achieves a high accuracy of 97% (29/30) for the segment-based evaluation. However, the actual first ISW of the signal is 15 seconds away from the predicted first ISW, a 15-second time difference that may not be acceptable in clinical scenarios.

Therefore, for the first time, we proposed time distance–based metrics to evaluate an automated PGES detection method. For a given signal recording *r*, we defined the *predicted time distance TD_r_* as the time difference between the predicted end time of PGES (or the predicted time of the first ISW) by the detection method and the actual end time of PGES (or the actual time of the first ISW) according to the expert annotations. A lower value of the time distance indicates a better performance of the PGES detection method.

On the basis of the predicted time distance, we further introduced the 5-second tolerance-based positive prediction rate (PPR_5s_) as another evaluation metric, as a 5-second time distance is acceptable according to clinical experts. Given a collection *R* of signal recordings for evaluation, we define *PPR_5s_* as follows:


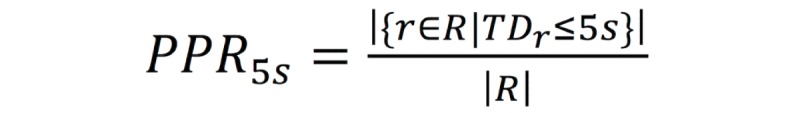


that is, the number of signal recordings whose predicted time distances are within 5 seconds divided by the total number of the signal recordings.

## Results

### Artifact Level

To evaluate the performance of our PGES detection method on EEG signals with different levels of artifacts, we categorized EEG signals into 4 levels: artifact-free, mild artifact, moderate artifact, and severe artifact. The domain expert manually reviewed the EEG signals and classified them into 4 levels according to the following criteria:

1. *Artifact-free*: No waveforms in any channels or abrupt waveforms of less than a second duration.
2. *Mild*: Abrupt waveforms in channels other than midline channels (Fz-Cz and Cz-Pz) that do not affect the midline channels or midline channels involved with abrupt waveforms of less than 1-second duration.
3. *Moderate*: One of the midline channels is involved, or both midline channels are involved with waveforms of less than 1-second duration.
4. *Severe*: Both midline channels have abrupt waveforms of more than 1-second duration. An expert may need to analyze all EEG chains, which include 19 EEG channels, or use video recordings to differentiate the artifacts from the brain-generated waveforms.

Among the 116 signal recordings in our dataset, 27 are artifact-free, 31 are with mild artifacts, 25 are with moderate artifacts, and 33 are with severe artifacts. We applied our PGES detection method to 4 groups of signal recordings with different levels of artifacts: only artifact-free (group A); artifact-free and mild artifact (group B); artifact-free, mild artifact, and moderate artifact (group C); and all signal recordings (group D).

### Cross-Validation

Cross-validation has been generally used for evaluating a model’s performance with low bias and variance. We applied 10-fold cross-validation 10 times to the 4 groups with varying artifact levels to evaluate our PGES detection method. For each group, we randomly separated the signal recordings into two parts each time, training set and testing set, and then calculated the evaluation metrics. Note that there was no overlap between the training set and testing set. For instance, in group D, which included all 116 signal recordings, 11 recordings were used as the testing data and the remaining 105 recordings were used as the training data in each fold. We repeated this procedure 10 times and used the average as the final evaluation result.

For the training set, we selected balanced numbers of PGES and ISW segments for each signal recording. Although every EEG recording was annotated by domain experts with the start of the first ISW, there existed EEG recordings missing the annotations of the end of the first ISW (as the onset of the first ISW is the most important). Therefore, for ISW signal segments, we selected up to 30-second signal (ie, 30 segments) after the onset of the first ISW as follows: if the duration of PGES (say *t* seconds) is less than 30 seconds, then we used *t* segments after the first onset of ISW; otherwise, we used 30 segments after the first onset of ISW. For the testing set, we used the entire 5 min of each signal recording to detect the first onset of ISW.

The average predicted time distance is 2.4 seconds for artifact-free signal recordings (group A) and 4.34 seconds for the group containing both artifact-free and mild artifact signal recordings (group B). As the artifact level increases, the average predicted time distance increases as well. The average predicted time distance is 7.54 seconds for the group containing artifact-free, mild artifact, and moderate artifact signal recordings (group C) and 7.84 seconds for all signal recordings (group D).

The *PPR_5s_* of our PGES detection method for each artifact group of signal recordings is as follows: *PPR_5s_* is 0.95 for artifact-free signal recordings (group A); 0.81 for the group containing both artifact-free and mild artifact signal recordings (group B); 0.73 for the group containing artifact-free, mild artifact, and moderate artifact signal recordings (group C); and 0.68 for all signal recordings (group D). It can be seen that *PPR_5s_* decreases as the level of artifacts increases.

**Figure 7:**
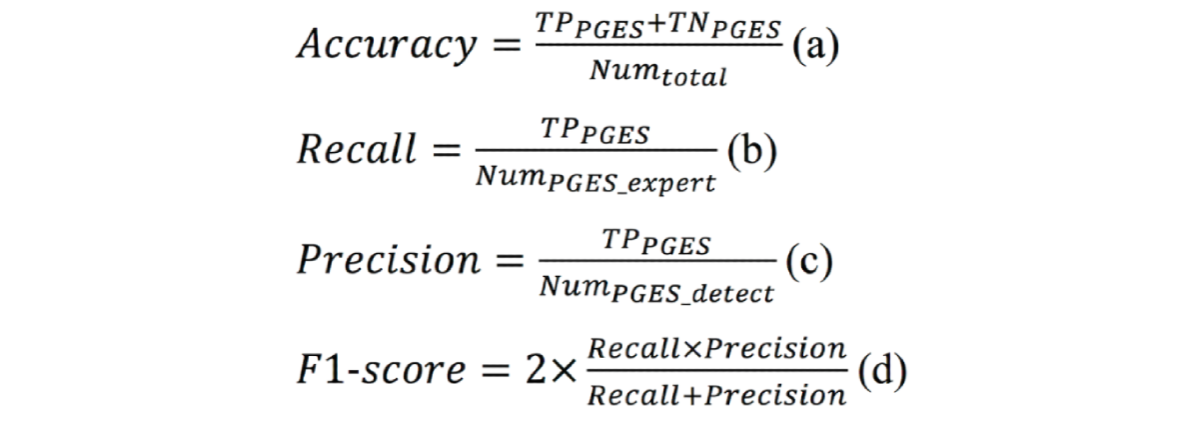
The definitions of segment-based evaluation metrics.

As a comparison, we also calculated the segment-based evaluation metrics to evaluate the performance of our PGES detection method for classifying individual signal segments, including accuracy, recall (*R_PGES_*), precision (*P_PGES_*), and F1-score (*F1_PGES_*), as defined in Figure 7, where *TP_PGES_* is the number of segments detected as PGES and labeled as PGES by experts, *TP_PGES_* is the number of segments detected as ISW and labeled as ISW by experts, *Num_PGES_expert_* is the total number of segments labeled as PGES by experts, *Num_PGES_detect_* is the total number of segments labeled as PGES by our detection method, and *Num_total_* is the total number of segments.

Table 1 shows the segment-based evaluation results of our method. The accuracy of each artifact group is over 0.92. On the other hand, the recall, precision, and F1-score for each group are over 0.95, 0.96, and 0.95, respectively.

**Table 1:** The traditional evaluation of postictal generalized electroencephalogram suppression (PGES) detection on each testing group.

Evaluation metric	Group A^a^	Group B^b^	Group C^c^	Group D^d^
Accuracy	0.94	0.94	0.94	0.92
Recall (R_PGES_)	0.95	0.96	0.97	0.95
Precision (P_PGES_)	0.98	0.98	0.97	0.96
F1-score (F1_PGES_)	0.97	0.96	0.97	0.95

^a^Only artifact-free signal recordings.

^b^Artifact-free and mild artifact signal recordings.

^c^Artifact-free, mild artifact, and moderate artifact signal recordings.

^d^All signal recordings.

## Discussion

### Principal Findings

We developed an automated PGES detection method based on EEG signals, which combined a random forest classifier and 3 correction rules. The main idea of our method was to leverage both signal features and the state transitions of brain activities. We evaluated the performance of our method using different artifact groups of signal recordings.

We reported both the segment-based evaluation results and the recording-based evaluation results. According to the segment-based evaluation results, our method achieved over 0.92 accuracy, 0.95 recall, 0.96 precision, and 0.95 F1-score for each artifact group. The results were consistent for each group, which indicates that our method performed well for classifying individual PGES signal segments (even for the group containing signal recording with severe artifacts).

However, as illustrated in Figure 6, the segment-based evaluation may not be able to demonstrate the actual PGES detection performance. In practical settings, the segment that was incorrectly detected as ISW would cause the wrong annotation of the PGES end time and result in an incorrect, significantly different PGES duration, which may mislead the risk assessment of SUDEP. Therefore, we introduced a way with direct practical relevance to evaluate automated PGES methods based on time distance, which is the time difference between the detected PGES period and the expert-annotated one.

On the basis of our recording and time distance–based evaluation, our PGES detection method achieved an average predicted time distance of 2.4 seconds and a PPR_5s_ of 0.95 for artifact-free EEG signals. For signals with artifacts, the performance of this method varies according to the level of artifacts. For signals with mild artifacts, our method achieved a PPR_5s_ of 0.81. However, as the number of signals with higher artifact levels (moderate) increased, the PPR_5s_ dropped to 0.73; for signals with all artifact levels (artifact-free to severe), it dropped to 0.68, and the average predicted time distance was 7.84 seconds. The artifact is the main challenge for PGES detection. To identify high-amplitude artifacts (severe level) that are not real brain activities, clinicians usually have to use different EEG patterns or even video recordings. In future work, we will focus on developing an approach for handling signals with high artifact levels (moderate and above). In particular, we plan to try dedicated artifact removal methods such as independent component analysis [46,47], regression analysis [48], and empirical method [49,50] to study whether such methods would help improving the performance of PGES detection.

Compared with the previous work [19], we used three additional types of features (time-domain features, wavelet-based features, and interchannel correlations) in the feature extraction step. For the classifier, we used random forest instead of boosting algorithms with logistic regression. We also introduced a new metric (*predicted time distance*) to evaluate an automated PGES detection method, and we reported evaluation results for both the segment-based method and our new metrics (no such evaluations were performed in the study by Theeranaew et al [19]).

### Conclusions

We presented an automated method that combines the benefits of random forest classifier and correction rules for PGES detection using multichannel EEG recordings. Features from temporal, frequency, wavelet, and cross-coherence analyses provided valuable information to characterize PGES and ISW. Confidence-based rules were leveraged to correct sudden changes of PGES states. We introduced a new evaluation method for assessing PGES detection performance with more practical relevance. The evaluation results indicated that our method achieved a PPR_5s_ of 0.95 for artifact-free EEG recordings. For EEG recordings with different artifact levels, the PPR_5s_ varied from 0.68 to 0.81. This study demonstrates that our combined random forest and rule-based approach can perform well in realistic settings for good quality EEG recordings.

## Abbreviations

AE: amplitude energy
CSR: Center for Sudden Unexpected Death in Epilepsy Research
EEG: electroencephalogram
EMU: epilepsy monitoring unit
GTCS: generalized tonic-clonic seizures
ISW: intermittent slow wave
NIH: National Institutes of Health
PGES: postictal generalized electroencephalogram suppression
PPR_5s_: 5-second tolerance-based positive prediction rate
SUDEP: sudden unexpected death in epilepsy
